# Evaluation of Immune Modulation by β-1,3; 1,6 D-Glucan Derived from *Ganoderma lucidum* in Healthy Adult Volunteers, A Randomized Controlled Trial

**DOI:** 10.3390/foods12030659

**Published:** 2023-02-03

**Authors:** Shiu-Nan Chen, Fan-Hua Nan, Ming-Wei Liu, Min-Feng Yang, Ya-Chih Chang, Sherwin Chen

**Affiliations:** 1 College of Life Science, National Taiwan University, Taipei 10617, Taiwan; 2 College of Life Science, National Taiwan Ocean University, Keelung 202301, Taiwan; 3 Taipei Hospital, Ministry of Health and Welfare, New Taipei City 242062, Taiwan

**Keywords:** β-glucan, *Ganoderma lucidum*, Reishi, innate and adaptive immune response, immune modulation, T-lymphocytes, Creatinine, randomized control trials

## Abstract

Fungi-derived β-glucan, a type of glucopolysaccharide, has been shown to possess immune-modulatory properties in clinical settings. Studies have indicated that β-glucan derived from *Ganoderma lucidum* (commonly known as Reishi) holds particular promise in this regard, both in laboratory and in vivo settings. To further investigate the efficacy and safety of Reishi β-glucan in human subjects, a randomized, double-blinded, placebo-controlled clinical trial was conducted among healthy adult volunteers aged 18 to 55. Participants were instructed to self-administer the interventions or placebos on a daily basis for 84 days, with bloodwork assessments conducted at the beginning and end of the study. The results of the trial showed that subjects in the intervention group, who received Reishi β-glucan, exhibited a significant enhancement in various immune cell populations, including CD3^+^, CD4^+^, CD8^+^ T-lymphocytes, as well as an improvement in the CD4/CD8 ratio and natural killer cell counts when compared to the placebo group. Additionally, a statistically significant difference was observed in serum immunoglobulin A levels and natural killer cell cytotoxicity between the intervention and placebo groups. Notably, the intervention was found to be safe and well tolerated, with no statistically significant changes observed in markers of kidney or liver function in either group. Overall, the study provides evidence for the ability of Reishi β-glucan to modulate immune responses in healthy adults, thereby potentially bolstering their defense against opportunistic infections.

## 1. Introduction

Throughout human existence, the immune system is continually challenged, leading to a heightened susceptibility to infections and abnormal reactions to antigens [1,2,3]. This prompts a keen interest in devising strategies to enhance the immune response through natural means, specifically by stimulating specific and non-specific immune responses [4,5]. As a matter of fact, the triggering of an immune response starts with the recognition of pathogen-associated molecular patterns, danger-associated molecular patterns, or both by a pattern recognition receptor (PRR) [6,7]. Moreover, a PRR agonist can serve as a vital component in activating specific (innate) and non-specific (adaptive) immune cells [8,9,10], which may become a prospective candidate to help managing an abberated immune response [11,12,13,14].

The search for natural interventions to positively modulate the immune response has led to a growing interest in nutraceuticals such as curcumin, elderberry, fungi and spirulina that have the potential to stimulate specific and non-specific immune responses [15,16,17,18]. Among these candidates, fungi-derived glucopolysaccharides known as β-glucan have shown to be particularly promising [19,20]. The Reishi mushroom, abundant in β-glucan, has a long-standing history in traditional medicine as an immune system supporter. Research has shown that Reishi may help to lower cholesterol levels by inhibiting the activity of a protein called PCSK9 [21], and also exhibit anti-inflammatory effects, which may help to improve gut health. In vitro studies have demonstrated that β-glucan are powerful immunomodulators that function through surface receptors such as dectin-1, complement receptor 3 (CR3), or toll-like receptors [22,23]. This recognition translates into intracellular signaling, subsequently orchestrating immune responses [23,24]. The practical applications of β-glucan are vast and include serving as a potential vaccine adjuvant for COVID-19, an anti-carcinogenic, an anti-pathogenic and a hypolipidemic [21,25,26,27]. They are also observed in trained immunity [28], supporting the development of memories for the innate immune system and serving as a secondary layer of protection against infection [29,30]. Furthermore, β-glucan derived from *Ganoderma lucidum*, also known as Reishi, has obtained GRAS status by the U.S Food and Drug Administration for use in a variety of daily consumable products [31]. Although several studies have confirmed the toxicity and efficacy of Reishi β-glucan [32,33,34], none have been performed in a randomized controlled trial setting with a pre-designated population group that can generalize the results. As a result, the current randomized controlled trial was conducted to reaffirm the safety and immunomodulatory effects of purified Reishi β-glucan among healthy adult volunteers.

## 2. Materials and Methods

### 2.1. Identity of the Intervention

β-glucan, a compound made up of D-glucose polymers, is mainly found in fungi, yeast and plants (grains) [34]. It is a vital component of the cell walls of most fungi and plants. The chemical structure of β-glucan polymers varies depending on the source, resulting in a diverse range of structural variations, including molecular weight, linkage pattern, degrees of branching, triple helical conformation, and water solubility [35]. Compared to other counterparts, mushroom derived β-glucan had a highly branched main chain with a combination of glycosidic 1,3, 1,4, and 1,6 β-linkages [36,37]. Several studies reported that β-glucan obtained from *G. lucidum* was confirmed by ^13^C NMR (Nuclear Magnetic Resonance) and FTIR (Fourier-transform infrared spectroscopy) spectroscopy with the most common chemical structure of β-glucan being β-1,3 backbone with different degrees of β-1,6 and/or β-1,4 branching [38,39].

In this study, the β-glucan was manufactured by Super Beta Glucan Inc. (Irvine, CA, USA). The *G. lucidum* strain used to produce β-glucan was confirmed via the BCRC (Bioresource Collection and Resource Center) repository. The test article used in the study had a composition of 91% carbohydrate, 1% fat, 1% protein, 2% ash, and 5% moisture. The final extract was analyzed using Megazyme’s mushroom and yeast β-glucan analytic kit and was found to contain approximately 75.2% β-glucan, which is commercially known as Reishi β-glucan/Immulink MBG^®^ (Super Beta Glucan Inc., Irvine, CA, USA) Reishi.

### 2.2. Study Population

#### 2.2.1. Eligible/Inclusion Criteria

The study was opened to adults of both genders between the ages of 18 and 55 who were in general good health, and were willing to participate in all study visits and procedures.

#### 2.2.2. Non-Eligible/Exclusion Criteria

The exclusion criteria implemented in this study were strictly followed to protect the safety and well-being of all participants and to make certain that the study population is consistent and that the intervention’s effects can be accurately determined by the study’s outcomes. Ineligible individuals include those unable to provide informed consent or comply with study protocols, those currently enrolled in other research studies, and smokers or users of tobacco products were not eligible to participate. Additionally, pregnant or nursing women, those with current respiratory illness, hypertension, peripheral vascular disease, eating disorders, or liver diseases, and individuals with a history of tuberculosis, hypothyroidism, hyponatremia, hyperthermia, or any significant hematological, endocrine, cerebrovascular, cardiovascular, coronary, pulmonary, renal, gastrointestinal, known immunocompromised/autoimmune disorder (including Addison’s disease, chronic fatigue syndrome, Crohn’s disease, celiac disease, human immunodeficiency virus, inflammatory bowel disease, psoriasis, and systemic lupus erythematosus), or neurological disease were also excluded. Furthermore, those who had undergone organ transplantation, were receiving immunosuppressant medication, had been diagnosed with cancer or diabetes mellitus, had a history of life-threatening allergic reactions, or were taking immune suppressive/modifying medications such as glucocorticoids, steroids, immunosuppressants, or any other immune related dietary supplements) were not eligible to participate. The study was conducted in Taipei, Taiwan in accordance with the Declaration of Helsinki, and was approved by an independent Institutional Review Board (IRB). It was recorded in the ISRCTN (International Standard Randomised Controlled Trials Number Registry, ISRCTN48306294; http://www.isrctn.com, accessed on 31 October 2022), and all eligible participants provided informed consent before the initial medical evaluation was conducted.

### 2.3. Study Design

The present investigation involved an in-depth examination of the effectiveness of Reishi β-glucan in a population of healthy adult volunteers, utilizing a randomized, double-blinded, placebo-controlled design. A thorough screening process was undertaken to carefully select and recruit a total of 126 eligible individuals who were then enrolled upon providing informed consent. The study protocol began with the administration of the intervention or placebo on Day 0, after each participant had undergone the collection of initial blood samples.

In this study, participants were randomly assigned to either an intervention group, receiving a daily dose of 200 mg of Reishi β-glucan in a capsule, or a placebo group, receiving 200 mg of dextrose monohydrate in a capsule, through a software-based random number generator at the time of enrollment. Participants were provided with an 84-day supply of their assigned capsules and instructed to take one daily, either before or while having breakfast, for the entire duration of the study. An interim safety check was conducted via phone call from Day 43 to Day 45, as deemed necessary by the Data and Safety Monitoring Board. At the conclusion of the 84-day administration period, blood samples were collected to analyze for primary outcomes, including the impact of Reishi β-glucan on various immune cells (CD3^+^, CD4^+^and CD8^+^ T-lymphocytes, NK cell counts/mediated cytotoxicity and serum IgA levels), as well as secondary outcomes, including various hematological biomarkers (alanine aminotransferase (ALT), aspartate aminotransferase (AST), Creatinine, red blood cell (RBC), hemoglobin (HB), hematocrit (HCT) and platelet counts) to determine the safety and tolerability of the intervention, in order to provide a comprehensive understanding of the effectiveness of Reishi β-glucan. 

### 2.4. Clinical and Laboratory Assays

To analyze for the primary outcome, a flow cytometric analysis of lymphocyte subsets was performed using the BD FACSCalibur™ flow cytometer (BD BioSciences, San Jose, CA, USA). Blood samples obtained from each participant were mixed with premixed monoclonal antibodies (mAbs)(BD BioSciences, San Diego, CA, USA) in two sets of antibody cocktails. The cocktails were prepared by combining 100 µL of whole blood sample with 10 µL of each mAbs including fluorescein isothiocyanate-conjugated (FITC) mouse anti-human CD3 mAb, phycoerythrin-conjugated (PE) mouse anti-human CD8 mAb, PE cyanin5-conjugated mouse anti-human CD4 mAb, allophycocyanin-conjugated (APC) mouse anti-human CD45 mAb, phycoerythrin-labeled anti-CD56 (B159), and fluorescein isothiocyanate–labeled anti-CD16 (3GB). The blood/antibody mixtures were then incubated for 15 min in a dark room under ambient conditions. Next, the red blood cells were lysed by adding 400 µL of FACS lysing solution (BD Biosciences, San Jose, CA, USA) for 15 min. Finally, 50 µL of CountBright^TM^ counting beads (Thermo Fisher Scientific, Waltham, MA, USA) were added to each staining mixture, and the samples were analyzed. Additionally, serum IgA levels were measured using a Human IgA (Immunoglobulin A) ELISA kit (eBioscience, San Diego, CA, USA) as per the manufacturer’s instructions.

The analysis of NK cell-mediated cytotoxicity was conducted utilizing the NK cell-mediated cytotoxicity assay (Invitrogen, San Diego, CA, USA). The procedure began with adjusting the target cell, YAC-1 (BCRC, Hsinchu, Taiwan), to a density of 1 × 10^6^/mL and staining with DiOC-18 at 37 degrees Celsius in a 5% CO_2_ environment for 20 min. The cells were then rinsed with PBS and suspended in RPMI 1640 medium to a density of 1 × 10^6^/mL. The NK cell and YAC-1 were mixed in ratios of 5:1, 10:1, and 20:1, with propidium iodide solution added to each mixture. The mixtures were then incubated at 37 degrees Celsius in a 5% CO_2_ environment for 2 h. Finally, using a flow cytometer (CyFlow Counter, Partec, Franklin Park, IL, USA), the cell mixtures were analyzed, with positive staining identifying viable target cells.

The secondary outcome was evaluated using standard hematology and blood chemistry techniques, including liver function tests (alanine ami-notransferase and aspartate aminotransferase), metabolic tests (Creatinine), and hematologic measurements (hemoglobin, hematocrit, and platelet counts).

### 2.5. Dose Determination

The dosage for the intervention was determined by referencing several studies. Wu et al. conducted an animal study using Reishi β-glucan as the intervention, administering a single daily dose of 8.5 mg/kg (low dose), 51 mg/kg (medium dose), and 170 mg/kg (high dose) to mice (*M. musculus*) for 42 consecutive days. These interventions were well-tolerated and led to notable changes in cytokine levels [40]. The human equivalent dose (HED) for these groups were calculated to be 0.69 mg/kg (low dose), 4.14 mg/kg (medium dose), and 13.82 mg/kg (high dose), respectively [41]. Additionally, a prior safety assessment study established a no-observed-adverse-effect level (NOAEL) of 2000 mg/kg bw/day [37], informing the calculation of a suitable daily dosage range of 48 to 1036 mg [41]. Furthermore, randomized controlled studies investigating the effects of baker’s yeast-derived β-1,3/1,6 glucans on circulating cytokines in relation to upper respiratory tract infections employed a dosage of 250 mg/day with good tolerability and statistically significant outcomes [42,43,44]. After considering the results from both the murine model and human randomized controlled trials, a daily dose of 200 mg/day was proposed, as it would elicit a dose-response association with the Reishi β-glucan on the proposed biomarkers while minimizing the risk of potential adverse reactions.

### 2.6. Interim Monitoring

The interim monitoring of this clinical study was handled by a data safety monitoring board (DSMB). The first interim monitoring took place at 42 days (6 weeks) from the first medical evaluation. As a result, DSMB reached a full consensus that no further interim monitoring was required due to the absence of adverse events reported. For this study, the stopping guidelines for futility were not considered.

### 2.7. Data Handling and Statistical Analysis

Data management was conducted according to the sponsor’s (SBG Biomedical Research, Los Angeles, CA, USA) recommended standard operation procedures, which followed the U.S Food and Drug Administration’s (USFDA) Good Clinical Practice (GLP) and Code of Federal Regulation 11 (CFR 11) [45]. Once the investigator determined that the data had met quality assurance standards, a series of restricted-access files were generated and sent to the biostatistician.

The significance of difference between groups was determined using a paired *t*-test, Student’s *t*-test, a Wilcoxon signed-rank test, and a Mann-Whitney U Test (Mann-Whitney-Wilcoxon Test). Differences were considered of statistical significance when the *p*-value was less than 0.05. Statistical analyses were generated using SPSS Version 25.0 (IBM, Chicago, IL, USA).

## 3. Results

**Figure 1:**
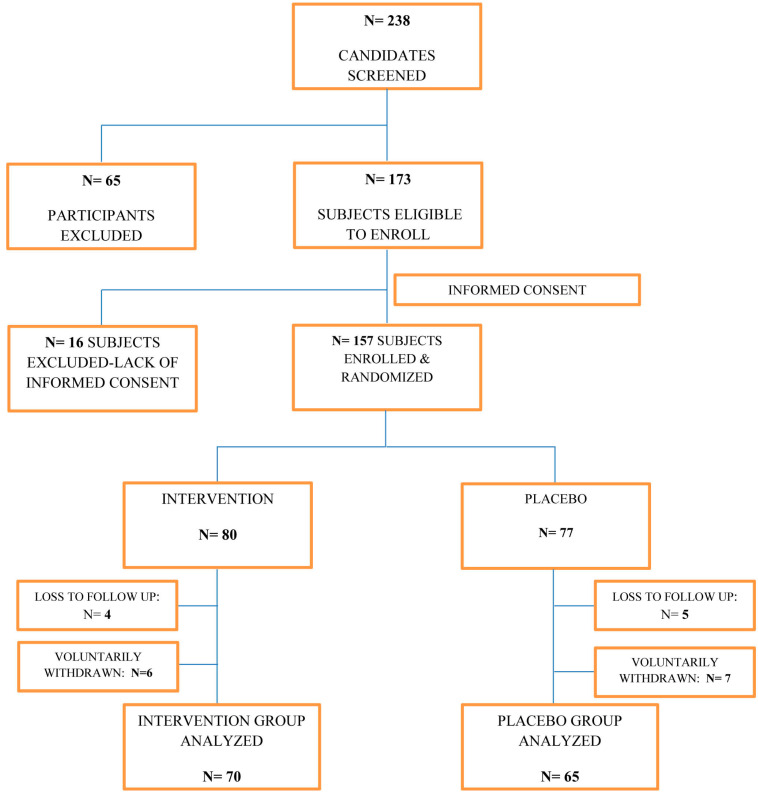
Study enrollment process flowchart with the numbers of subjects analyzed in each group.

The present study initially screened a total of 238 individuals, with 65 being disqualified due to exclusion criteria and an additional 16 being unable to participate due to incomplete informed consent. Ultimately, 157 participants were randomized and assigned to either the intervention group (*n* = 80) or the placebo group (*n* = 77). Of these, 70 participants in the intervention group and 65 in the placebo group were able to successfully complete the study and were included in the final analysis. The reasons for dropout included loss to follow-up (four in the intervention group and five in the placebo group) and voluntary withdrawal (six in the intervention group and seven in the placebo group) (Figure 1). The average age of participants in the intervention and placebo groups, along with their respective standard deviations, was 37.72 ± 7.36 and 39.11 ± 7.52 years, respectively. There was no statistically significant difference in gender distribution between the groups (54.2% males in the intervention group and 58.4% males in the placebo group; *p* = 0.85, chi-square test). A summary of baseline outcomes for the participants can be found in Table 1.

### 3.1. Primary Outcomes

After 12 weeks, the examination of the primary outcomes between the intervention and placebo group revealed a statistically significant alteration in immune-related parameters. The intervention group displayed a statistically significant enhancement in each parameter as compared to the placebo group, as demonstrated in Table 2. Furthermore, the analysis showed a statistically significant difference in NK cell-mediated cytotoxicity between the intervention and the placebo group after 12 weeks (*p* = 0.026), as outlined in Table 2, when compared to the baseline measurements presented in Table 1.

### 3.2. Secondary Outcomes

After conducting a thorough analysis of the data collected throughout the course of the 12-week study, it was determined that there were no statistically significant differences observed between the intervention group and the placebo group with regard to any of the secondary outcome measures.

### 3.3. Intra-Group Analyses

**Figure 2:**
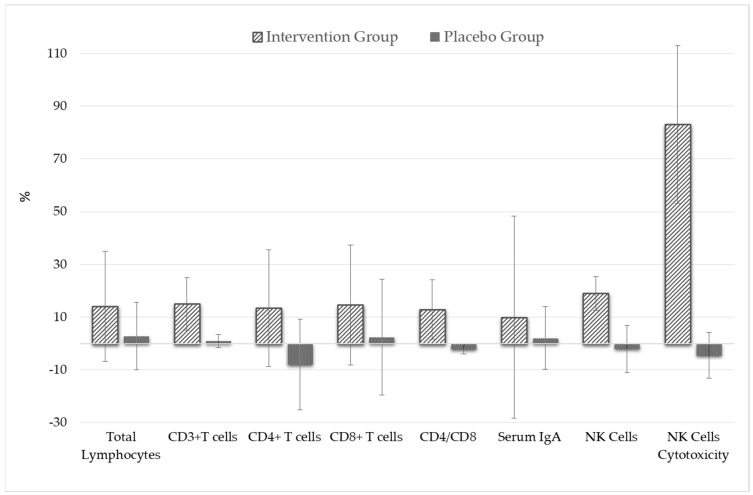
Percentage changes in primary outcome between baseline and post-intervention measurements (after 12th week).

An intra-group analysis was conducted to evaluate the impact of the intervention on primary and secondary outcomes by comparing the percentage changes from baseline to post-12 weeks between the intervention and placebo groups. The results, presented in Figure 2, reveal a statistically significant difference in a number of serum biomarkers between the intervention and placebo group, including Total lymphocyte count (14.1 ± 20.9% vs. 2.8 ± 12.8%; *p* = 0.002); absolute CD3^+^ T-cell counts (15.0 ± 9.9% vs. 1.0 ± 2.5%; *p* = 0.002); absolute CD4^+^ T-cell counts (13.4 ± 22.2% vs. −8.0 ± 17.2%; *p* = 0.0001), absolute CD8^+^ T-cell counts (14.6 ± 22.8% vs. 2.4 ± −22%; *p* = 0.033), CD4-to-CD8 ratio (12.9 ± 11.2% vs. −2.2 ± 1.7%; *p* = 0.0001), serum IgA concentration (10.0 ± 38.3% vs. 2.1 ± 11.8%; *p* = 0.031), NK cell counts (19.5 ± 6.4% vs. −2.0 ± 8.9%; *p* = 0.0001) and NK cell cytotoxicity (83.1 ± 30.0% vs. −4.5 ± −8.7%; *p* = 0.0001). Furthermore, the secondary outcome measurements revealed no statistically significant differences in any biomarkers between the intervention and placebo groups, indicating that the intervention was safe and well-tolerated among healthy adults when taken daily for the entire study.

## 4. Discussion

The recent emergence of infectious diseases as a global pandemic has underscored the imperative to create interventions that can effectively regulate both innate and adaptive immune responses. A plethora of studies have demonstrated that natural substances can stimulate pattern recognition receptors (PRRs), which are crucial components of the non-specific (innate) immunity that is needed to activate antigen-presenting cells (APCs). These APCs interact with T-cells, thus linking innate and adaptive immune responses.

*Ganoderma lucidum*, commonly known as the Reishi mushroom, has been acknowledged for its health benefits in traditional medicine for centuries. It is known for its ability to lower lipid levels through PCSK9 inhibition [21], its anti-metastatic and anti-carcinogenic effects, as well as its ability to modulate the immune response against pathogenic infections [46,47]. One of its key constituents, β-glucan, is a known PRR agonist that can effectively regulate immune responses in both in vitro and in vivo settings [47]. However, the immunomodulatory properties of Reishi β-glucan have yet to be confirmed in a randomized controlled trial, which is the primary motivation behind the current study.

The key findings from this study included that Reishi β-glucan induced statistically significant modifications of the CD3^+^, CD4^+^, CD8^+^ T-lymphocytes and NK cells, as well as elicited a statistically significant increase in serum IgA concentration in the intervention group versus placebo. The results concurred with a similar finding by Henao et al., where β-Glucan was provided to adolescents via a yogurt mixture [48]. Additionally, Reishi β-glucan also stimulated a statistically significant NK cell-mediated cytotoxicity by 83.1% in the intervention group as compared to the placebo.

Previous studies have demonstrated that β-glucan derived from mushrooms and yeast (*Saccharomyces cerevisiae*) possess the ability to stimulate the production of monocytes, macrophages, dendritic cells, and lymphocytes in both in vitro and in vivo settings [49,50]. The current study, however, goes further by providing evidence that Reishi β-glucan, when taken as an intervention, can also lead to an expansion of NK cells and T-lymphocyte counts. This finding supports our initial hypothesis that, given its similar molecular structure to other β-glucans used in previous research [49,50,51,52], Reishi β-glucan possesses a comparable ability to modulate both innate and adaptive immune responses.

The immune system has the unique ability to generate memory responses after being exposed to infections or vaccinations, which enables swift and heightened immune responses to secondary infections. This capacity, referred to as innate immune memory or trained immunity, is not restricted to a specific pathogen, and can enhance protection against repeated pathogenic invasions [28]. This ability of the immune system to generate memory responses allows it to quickly recognize and respond to previously encountered pathogens. This is important, as it can help to prevent the progression of the disease and limit the spread of the pathogen, thus minimizing the damage caused by the infection. In multiple studies, β-glucan was highly capable of inducing trained immunity, which exhibited enhanced immune responses upon re-stimulation with various microbial ligands responsible for protection against opportunistic infections [29,30]. Although in rodent models certain carbohydrates and polysaccharides were previously known to induce immune responses independently of T-cells, nevertheless, polysaccharides such as β-glucan could elicit a similar response via activating CD4^+^ and CD8^+^ T-cells through the MHC-I and MHC-II endocytic pathway [53]. Via an enhancement of trained immunity as well as the T-lymphocyte activation, β-glucan may enhance immunity and reinforce natural protections among healthy adult populations [51,54,55].

IgA plays an important role in the regulation of immune responses. Results from the animal studies suggested that treatments of β-glucan derived from *G. lucidum* could modulate serum immunoglobulin production such as IgA and IgG1 in vivo [40]. In another pilot study, the dietary intake of soluble β-glucan led to an increase in salvia IgA concentration [56]. The present investigation posits that the ingestion of β-glucan may elicit the PRR-dependent activation of immune cells, as opposed to inciting antigen-specific B-cell responses that result in a substantial secretion of IgA. This may be attributed to the ability of serum IgA to bind to Fcα-receptor I (FcαRI) expressed by monocytes, neutrophils, and dendritic cells (DCs), thereby triggering pro-inflammatory responses [57]. However, further research is necessary to fully understand and validate the underlying mechanisms of Reishi β-glucan and its impact on serum IgA production.

On the other hand, a comprehensive serum cytokine analysis was not proposed for the study. Unlike T- and NK cell activities, which can be enduring, cytokine secretion was a short-lived, self-limited event and its synthesis was commenced, as a result of the cellular activation, by new gene transcriptions. The transcriptional activation was brief, and the messenger RNAs encoding most cytokines were unstable, which made cytokine synthesis a transitory event as well. This phenomenon would ultimately create multiple challenges if a plan analyzing cytokines was in place during the trial. Second, most cytokine productions are local and act close to where they are produced, either on the same or on a nearby cell. And when produced in large amounts, cytokines may enter the circulation and function away from the production site; on the contrary, they may not be detected even if being produced. Finally, cytokines were known to be pleiotropic and redundant, which refers to the fact that multiple cytokines may have the same functional effects. This would also create complexity in our plan to evaluate the effectiveness of the intervention versus the primary and secondary outcome.

The innate immune system plays a pivotal role in the identification and eradication of viral and bacterial invaders. This is achieved through the detection of pathogen-associated molecular patterns and the activation of complement binding [55]. Currently, researchers are investigating new strategies that focus on manipulating the innate immune system’s response to pathogens in order to enhance resistance to infection. This approach holds great promise, as it has the potential to provide broad-spectrum protection against a wide range of pathogens without the need for a specific diagnosis, and may enable the prevention and treatment of infection. Previous studies have shown that pre-treatment with β-glucan from *G. lucidum* may augment NK cell-mediated cytotoxicity in vitro [40], which is believed to be mediated by the direct binding to the NKp30 activating receptor [58]. The current study has revealed a statistically significant increase in NK cell counts, as well as an 83.1% increase in cytotoxicity in the intervention group as compared to the placebo group. NK cells are a crucial component of the innate immune system, serving as the body’s first line of defense against infections and diseases, including cancer. Their cytotoxicity is a unique and instantaneous mechanism that does not require prior antigen-priming, but rather is regulated by a complex network of receptors [57,58,59]. These findings suggest that the regular dietary intake of Reishi β-glucan can bolster innate immunity and provide stronger protection through cellular cytotoxicity in response to opportunistic viral infections and malignancies among healthy individuals.

Finally, the results of the secondary outcome indicate that the regular dietary intake of Reishi β-glucan for a period of 12 weeks was well tolerated among healthy participants aged between 18 and 55 years. There were no reported adverse side effects, and no statistically significant changes were found between the intervention and placebo groups in the secondary outcome, which is in line with the predictions made from a previous subchronic toxicity study in rodents [37]. In conclusion, the current randomized controlled trial confirms that Reishi β-glucan, a known PRR agonist, can effectively modulate the immune system by activating both innate and adaptive immune responses. However, further investigations are needed to fully understand its therapeutic mechanisms, such as its pharmacokinetics and bioavailability. This could include exploring the use of nanoparticles for targeted delivery [60] and measuring its effects on other components of the immune system, such as phagocytic and anti-microbial peptide activities. Additionally, it would be important to conduct further studies to understand the optimal dosage and duration of Reishi β-glucan intake for maximum efficacy, as well as its potential interactions with other medications.

## 5. Conclusions

The current randomized controlled trial has confirmed that Reishi β-glucan, a known PRR agonist, can effectively modulate the immune system by activating both innate and adaptive immune responses. This enhancement of immunity provides robust protections in the face of opportunistic pathogenic infections and cellular malignancies among healthy individuals. Additionally, the trial also found that the regular dietary intake of Reishi β-glucan for a period of 12 weeks was well tolerated with no reported adverse side effects.

## Figures and Tables

**Table 1:** Baseline analysis of primary and secondary outcomes categorized by groups prior to interventions/placebo administration.

Parameter	Intervention Group (*n* = 70)	Placebo Group (*n* = 65)	*p* Value *	Statistically Significant Difference between Study Groups
	Mean ± SD	Mean ± SD		
**Primary outcome**				
Total Lymphocytes (cells/µL)	1932.3 ± 508.5	1820.6 ± 438.2	0.557	*p* > 0.05
CD3^+^ T lymphocytes (cells/µL)	1389.2 ± 392.0	1285.5 ± 323.1	0.615	
CD4^+^ T lymphocytes (cells/µL)	1086.9 ± 374.5	1018.7 ± 294.8	0.928	
CD8^+^ T lymphocytes (cells/µL)	782.2 ± 260.7	763.0 ± 240.3	0.619	
CD4/CD8 cell ratio	1.39 ± 0.62	1.33 ± 0.57	0.864	
Serum IgA (mg/dL)	262.3 ± 112.8	247.1 ± 121.2	0.105	
NK Cells (cells/µL)	285.5 ± 143.1	278.9 ± 130.7	0.093	
NK Cells Cytotoxicity (%)	36.8 ± 9.5	37.5 ± 9.1	0.431	
**Secondary Outcome**				
AST (mg/dL)	24.8 ± 8.6	23.2 ± 7.9	0.503	*p* > 0.05
ALT (mg/dL)	15.2 ± 6.3	14.9 ± 5.8	0.102	
Creatinine (mg/dL)	0.86 ± 0.08	0.83 ± 0.07	0.114	
RBC (10^6^/µL)	4.75 ± 0.41	4.93 ± 0.54	0.352	
HB (g/dL)	14.52 ± 2.01	14.70 ± 1.94	0.527	
HCT (%)	43.31 ± 4.25	44.12 ± 3.87	0.738	
Platelet Counts (10^3^/µL)	264.82 ± 72.13	259.15 ± 68.42	0.570	

Abbreviation: ALT, alanine aminotransferase; AST, aspartate aminotransferase; IgA, immunoglobulin A; NK, natural killer; Ig, immunoglobulin; RBC, red blood cell; HB, hemoglobin; HBC, hematocrit. * *p* < 0.05 = statistically significant.

**Table 2:** Post-intervention analysis of the primary and secondary outcome categorized by groups, following 12 weeks of interventions/placebo administration.

Parameter	Intervention Group (*n* = 70)	Placebo Group (*n* = 65)	*p* Value *	Statistically Significant Difference between Study Groups
	Mean ± SD	Mean ± SD		
**Primary outcome**				
Total Lymphocytes (cells/µL)	2206.3 ± 614.5	1872 ± 494.3	0.012 *	Yes, increase
CD3^+^ T lymphocytes (cells/µL)	1598.8 ± 431.1	1299.4 ± 331.2	0.001 *	Yes, increase
CD4^+^ T lymphocytes (cells/µL)	1233.3 ± 457.8	936.7 ± 243.8	0.003 *	Yes, increase
CD8^+^ T lymphocytes (cells/µL)	896.9 ± 320.3	754.9 ± 187.2	0.025 *	Yes, increase
CD4/CD8 cell ratio	1.57 ± 0.69	1.30 ± 0.58	0.036 *	Yes, increase
Serum IgA (mg/dL)	288.7 ± 156.1	252.3 ± 135.6	0.048 *	Yes, increase
NK Cells (cells/µL)	341.2 ± 152.3	284.6 ± 142.4	0.045 *	Yes, increase
NK Cells Cytotoxicity (%)	67.4 ± 12.4	35.8 ± 8.3	0.001 *	Yes, increase
**Secondary Outcome**				
AST (mg/dL)	25.6 ± 9.3	23.7 ± 8.5	0.426	No, unchanged
ALT (mg/dL)	15.4 ± 6.4	15.6 ± 6.0	0.124	No, unchanged
Creatinine (mg/dL)	0.83 ± 0.1	0.90 ± 0.12	0.325	No, unchanged
RBC (10^6^/µL)	4.68 ± 0.73	4.98 ± 0.67	0.311	No, unchanged
HB (g/dL)	14.12 ± 3.40	13.94 ± 2.73	0.570	No, unchanged
HCT (%)	44.13 ± 5.03	43.98 ± 4.68	0.694	No, unchanged
Platelet Counts (10^3^/µL)	258.63 ± 63.52	249.31 ± 59.45	0.627	No, unchanged

* *p* < 0.05 = statistically significant.

## Data Availability

The data presented in this study are available on request from the corresponding author. The data are not publicly available due to privacy and confidentiality.
